# Supplementary material for: Genetic comparisons of interleukin-17 reveal a framework for complex signaling evolution
Source: bioRxiv. 2026 Apr 14:2026.04.13.718218. Preprint. [Version 1] doi: 10.64898/2026.04.13.718218 (PMC13105070; doi:10.64898/2026.04.13.718218)
Supplement: Supplement 3 [file media-3.zip › Supplemental files for paper/ERC/IL17xIL17_comparisons/IL17F_IL17RA.pdf]

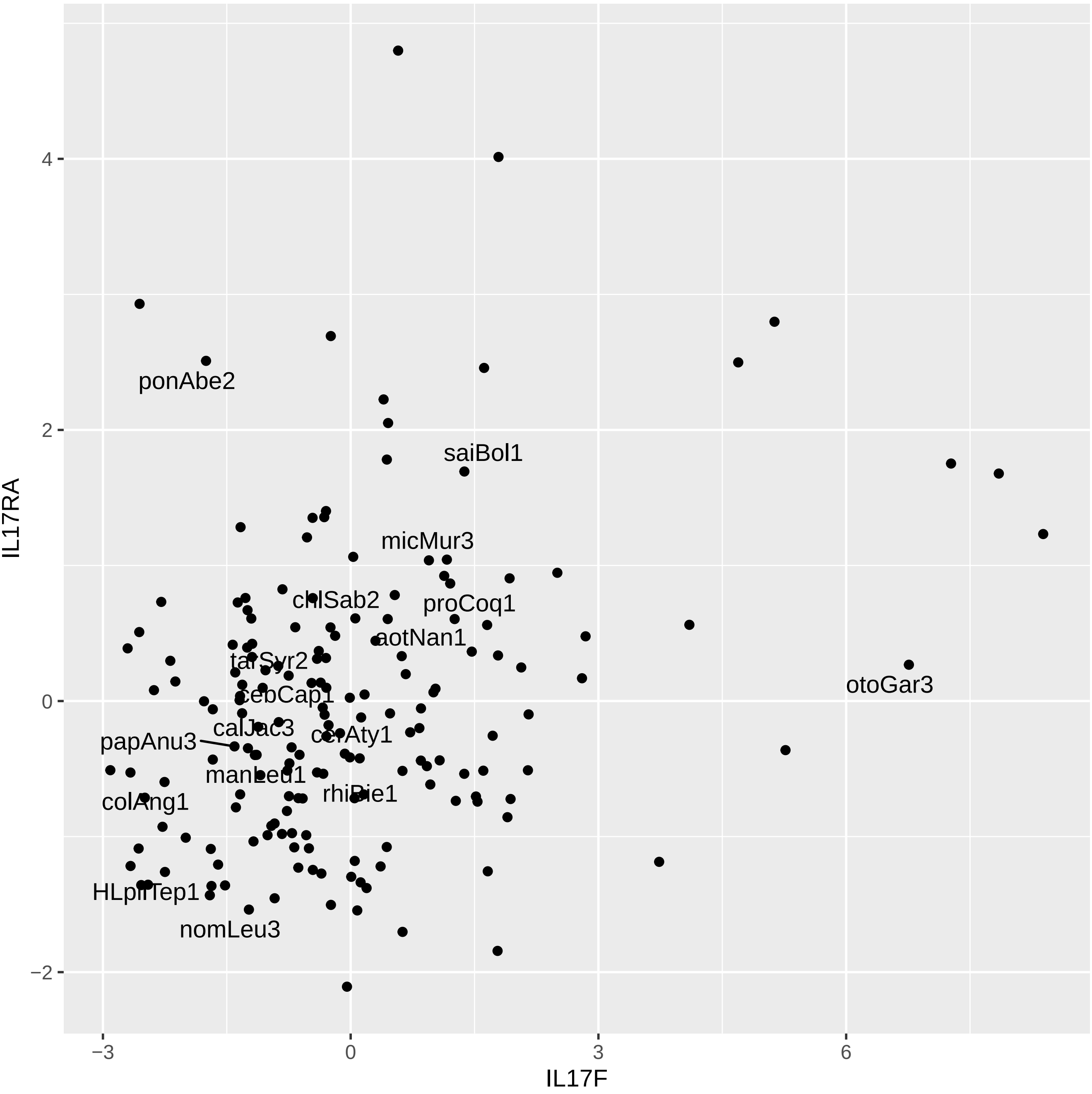
ponAbe2
saiBol1
micMur3
chlSab2
proCoq1
aotNan1
tarSyr2
cebCap1
calJac3
papAnu3
cerAty1
manLeu1
rhiBie1
colAng1
HLpilTep1
nomLeu3
otoGar3
IL17RA
IL17F
4
2
0
−2
−3
0
3
6
